# A Novel Small Molecular Inhibitor of DNMT1 Enhances the Antitumor Effect of Radiofrequency Ablation in Lung Squamous Cell Carcinoma Cells

**DOI:** 10.3389/fphar.2022.863339

**Published:** 2022-03-23

**Authors:** Yuan-Yuan Liu, Cheng-Zhi Ding, Jia-Ling Chen, Zheng-Shuai Wang, Bin Yang, Xiao-Ming Wu

**Affiliations:** ^1^ Department of Thoracic Surgery, He Nan Provincial Chest Hospital, Zhengzhou, China; ^2^ Department of Traditional Chinese Medicine, Zhengzhou Xinhua Hospital of Traditional Chinese Medicine, Zhengzhou, China; ^3^ Department of Hepatology, The Fifth Medical Center of Chinese PLA General Hospital, Beijing, China

**Keywords:** radiofrequency ablation, lung squamous cell carcinoma, DNA methyltransferase 1, small molecular inhibitor, Notch pathway

## Abstract

Radiofrequency ablation (RFA) is a relatively new and effective therapeutic strategy for treating lung squamous cell carcinomas (LSCCs). However, RFA is rarely used in the clinic for LSCC which still suffers from a lack of effective comprehensive treatment strategies. In the present work, we investigate iDNMT, a novel small molecular inhibitor of DNMT1 with a unique structure. In clinical LSCC specimens, endogenous DNMT1 was positively associated with methylation rates of miR-27-3p′s promoter. Moreover, endogenous DNMT1 was negatively correlated with miR-27-3p expression which targets PSEN-1, the catalytic subunit of γ-secretase, which mediates the cleavage and activation of the Notch pathway. We found that DNMT1 increased activation of the Notch pathway in clinical LSCC samples while downregulating miR-27-3p expression and hypermethylation of miR-27-3p′s promoter. In addition of inhibiting activation of the Notch pathway by repressing methylation of the miR-27-3p promoter, treatment of LSCC cells with iDNMT1 also enhanced the sensitivity of LSCC tumor tissues to RFA treatment. These data suggest that iDNMT-induced inhibition of DNMT-1 enhances miR-27-3p expression in LSCC to inhibit activation of the Notch pathway. Furthermore, the combination of iDNMT and RFA may be a promising therapeutic strategy for LSCC.

## 1 Introduction

The majority of lung tumors are either non-small cell lung cancers (NSCLCs) or small cell lung cancers. Other types of lung tumors include lung lymphomas or lung metastases from other tumors. NSCLC is the most common and frequent pathological subtype of lung cancer (Baas et al., 2021; Felip et al., 2021; Thai et al., 2021). While most research on NSCLC has focused the subtypes known as lung adenocarcinomas (LUADs), there is still a lack of knowledge and research about lung squamous cell carcinomas (LSCCs) (Acker et al., 2021; Hu et al., 2021; Nicholson et al., 2022). Percutaneous radiofrequency ablation (RFA) is generally an important treatment strategy in cancer therapy, but it is also an important strategy for minimally invasive treatment of tumors (Xie et al., 2017; Feng et al., 2018a; Huber et al., 2021; Kuo et al., 2021) because RFA has several advantages for LSCC treatment, and it has been the source of recent research interests. The first advantage of RFA therapy lies in the fact that RFA may directly induce injury of LSCC lesions/tumors, thus reducing the risk of injury to normal surrounding tissue, including the respiratory tract (Cazalas et al., 2021). A second advantage lies in the fact that the use of RFA guided using a bronchoscope is a safer and more efficient method for treating LSCC than that of percutaneous RFA for visceral organ tumors, such as HCC (hepatocellular carcinoma) (Olive et al., 2021). Current studies have shown that combination treatment with RFA and antitumor drugs can achieve superior antitumor effects (Ding et al., 2020; Jin et al., 2021; Oyama et al., 2021). Although RFA treatment of LSCC has many advantages, it faces many challenges: the temperature of RFA treatment is too high, and if the treatment time is too long, it may cause damage to the surrounding tissue (such as the trachea or bronchi). However, if the RFA treatment temperature is lower and the time is shorter, incomplete ablation may occur. Therefore, RFA may be a significant game changer in future LSCC treatment regimens, and it is of great significance to research and develop new RFA treatment strategies to achieve milder RFA conditions (lower temperature and shorter time), that is, to achieve equal or superior antitumor activation.

While the Notch signaling pathway plays an important role in normal cell carcinogenesis, it is also an important regulator of cell survival and antiapoptotic pathways (Zhang et al., 2017; Farooqi et al., 2020; Jia et al., 2021a). Moreover, the Notch signaling pathway is the core stress and injury response in malignant tumor cells (Zhang et al., 2021a; Shu et al., 2021; Weir et al., 2021). For human malignant tumor cells, radiation (i.e., ionizing radiation (IR)) as well as other antitumor treatment strategies induces damage and acts as stress factors. IR itself, as well as other factors, can activate the Notch pathway (Kang et al., 2013; Grassi et al., 2020; Fang et al., 2021). The activated Notch pathway can increase cell proliferation by inducing cell EMT and upregulating factors related to cell survival and antiapoptotic processes (Zhang et al., 2021b; Pu et al., 2021; Yazaki et al., 2021), eventually leading to increased resistance to antitumor treatment strategies (Zhang et al., 2021b; Pu et al., 2021; Yazaki et al., 2021). At present, there is no clear clinical cohort study on the relationship between the Notch pathway and RFA, but in addition to radiotherapy and chemotherapy, EMT and other mechanisms have also been found to be closely related to poor prognosis, incomplete ablation, and recurrence of interventional therapies such as RFA and TACE (Zhang et al., 2017; Kuo et al., 2021). This suggests that inhibiting the EMT process in LSCC tissues by inhibiting the activity of the Notch pathway is an ideal strategy to achieve RFA sensitization. Activation of the Notch pathway is mainly due to the two-step cleavage of the Notch protein by a disintegrin and metalloproteinase (ADAMs) and γ-secretase. This leads to the subsequent release of NICD which translocates from the cytoplasm into the nucleus to mediate EMT expression, and cell pro-survival–antiapoptosis-related factors (Zhang et al., 2018; Ferreira and Aster, 2021; Sezin et al., 2022). Given that the first cleavage step is mediated by ADAMs, including ADAM17 and ADAM10, pharmacological targeting of either could result in compensatory effects. However, because the second cleavage step is mediated by γ-secretase alone, pharmacological inhibition of this protein may be a promising approach to impede activation of the Notch signaling pathway, thus avoiding any compensatory effect between ADAM17 with ADAM10 (Zhao et al., 2021). Therefore, γ-secretase may be a promising drug target for inhibiting the Notch pathway, and it may also improve the efficacy of antitumor therapy.

MicroRNAs (miRNAs and miRs) are a large series of 18–25 nucleotide (nt) long noncoding RNAs that are transcribed by RNA polymerase II (Wang et al., 2020; Chong et al., 2021; Jin, 2021). Increasing evidence has shown that hypermethylation in the 5′–C–phosphate–G–3 (5′-CpG-3′) islands of the promoter region of miRs, functioning as tumor suppressors by DNMT-1, often mediates activation of the proliferation or drug-resistance-related pathways (Wong, 2020; Wong, 2021; You et al., 2021). Therefore, small molecular inhibitors of DNMT-1 may be potentially promising antitumor therapies (Wang et al., 2021a). In the present work, we show that miR-27-3p acts on the 3′UTR of PSEN-1 to inhibit PSEN-1 expression to reduce Notch pathway activity. In LSCC tissues, incomplete RFA can upregulate the expression of factors related to epithelial–mesenchymal transition and pro-survival/antiapoptosis downstream of the Notch pathway. Treatment with iDNMT inhibits DNMT-1-mediated hypermethylation of the miR-27-3p promoter region, upregulates miR-27-3p expression, and inhibits Notch pathway activity by repressing PSEN-1 expression and ultimately upregulates the killing effect of RFA on LSCC.

## 2 Materials and Methods

### 2.1 Clinical Specimens, Cell Lines, and Agents

A total of 25 LSCC clinical specimens were kindly gifted by Prof. and Dr. Wei Zhou (Beijing Hospital, Beijing, China) (Zhou et al., 2021). The use of human samples, including clinical tumor tissue samples and cell lines, were reviewed and approved by the Ethics Committees of Henan Provincial Chest Hospital. The collection of clinical specimens was approved by the Ethics Committee of Beijing Hospital with written informed consent provided by all patients (approval ID: 2019BJYYEC-125-02). The protocol and the experimental design of this work were approved by Henan Provincial Chest Hospital. The H226 LSCC cell line was also gifted by Prof. and Dr. Wei Zhou of Beijing Hospital (Zhou et al., 2021). The vectors of hsa-pre-miR-27, PSEN-1, and NICD were gifted by Prof. and Dr. Hua Yang (Hebei University, Baoding, Hebei Province, China) (Zhao et al., 2021). The expression vectors for DNMT-1 and siDNMT1 were gifted by Prof. and Dr. Qiyu Jiang (Institute of Infectious Disease, Department of Infectious Disease, The Fifth Medical Center of Chinese PLA General Hospital, Beijing, China) (Ma et al., 2020).

### 2.2 Analysis of Methylation Rates by BSP-NGS and the qPCR

The genomic DNA samples from the clinical specimens, tumor tissues, and cultured cells were extracted and isolated using the DNeasy Blood & Tissue Kit (Cat No. 69504; QIAGEN, Hilden, Germany) and treated with the EpiTect Bisulfite Kit (Cat No. 59104; QIAGEN) according to the manufacturer’s instructions. Next, the polymerase chain reactions (PCRs) were performed using DNA samples and a high-fidelity polymerase, Platinum II Hot-Start PCR Master Mix (Cat No. 14000012; Thermo Fisher Scientific), to amplify the selected promoter region (chr19: 13836440-13838517, negative chain [-], the selected promoter region of has-pre-miR-27) of miR-27 with CpG islands (chr19: 13,835,599-13,835,325, negative chain [-]). We used the following primers for this word: sense sequence, 5′-GGYGATAGAGTGAGATTTTG-3′; antisense sequence 5′-AAATTTTCAAAACCCRATACA-3′. Following PCR experiments, the products were directly sequenced by using Ion Torrent PGM, and the methylation rates were calculated as previously described (Ma et al., 2020; Wang et al., 2021b; He et al., 2021).

For quantitative PCR (qPCR) analysis, the total RNA was extracted from the clinical specimens, LSCC cells, or subcutaneous tumor tissues as described by Ma *et al.* (2020) before reverse transcription to generate cDNA. The expression of DNMT1, Notch-related factors, and has-pre-miR-27 was examined by real-time quantitative fluorescence PCR (RT-PCR/qPCR) (Ma et al., 2016; Yang et al., 2020; Du et al., 2021) with β-actin as the loading control. The following primers were used in these qPCR experiments: 1) E-cadherin (CDH1), sense 5′-CTC​CTG​AAA​AGA​GAG​TGG​AAG​TGT-3′; antisense 5′-CCG​GAT​TAA​TCT​CCA​GCC​AGT​T-3′; 2) N-cadherin (CDH2), sense 5′-CCTGGAT CGCGAGCAGATA-3′; antisense 5′-CCA​TTC​CAA​ACC​TGG​TGT​AAG​AAC-3′; 3) vimentin, sense 5′-ACC​GCA​CAC​AGC​AAG​GCG​AT-3′; antisense 5′-CGA​TTG​AGG​GCT​CCT​AG CGGTT-3′; 4) DNMT1, sense 5′-GTG​GTG​GTG​GAT​GAC​AAG​AAG​T-3′, antisense 5′-AGG​CTC​CCC​GTT​GTA​GGA-3’; 5) PSEN-1, sense 5′-CCA​TAT​TGA​TCG​GCC​TGT​G-4′; antisense 5′-GAA​GGG​CTG​CAC​GAG​ATA​AT-3′; 6) BCL2, sense 5′-GAT​CGT​TGC​CTT​ATG​CA TTTGTTTTG-3′; antisense, 5′-CGG​ATC​TTT​ATT​TCA​TGA​GGC​ACG​TTA-3′; 6) NICD, sense 5′-CCG​ACG​CAC​AAG​GTG​TCT​T-3′; antisense 5′-GTC​GGC​GTG​TGA​GTT​GAT​GA-3; 7) survivin (BIRC2), sense 5′-ACA​TGC​AGC​TCG​AAT​GAG​AAC​AT-3′; antisense 5′-GATTCCCA ACACCTCAAGCCA-3′; 8) cIAP-1 (BIRC3), sense 5′-GTG​TTC​TAG​TTA​ATC​CTG​AGC​AGC TT-3’; antisense 5′-TGG​AAA​CCA​CTT​GGC​ATG​TTG​A-3′; 9) cIAP-2 (BIRC5), sense 5′-CAA​GGA​CCA​CCG​CAT​CTC​T-3′; antisense 5′-AGC​TCC​TTG​AAG​CAG​AAG​AAA​CA-3’; (10) β-actin, sense 5′-CAC​CAT​TGG​CAA​TGA​GCG​GTT​C-3′; antisense 5′-AGGTCTTTGCGGA TGTCCACGT-3′; 11) miR-27a-3p (has-pre-miR-27), loop primer 5′-GTC​GTA​TCC​AGT​GCG​TG TCG​TGG​AGT​CGG​CAA​TTG​CAC​TGG​ATA​CGA​CGC​GGA​A-3′; sense 5′-GGCTAAGTTC CGCGTCGTAT-3′; antisense 5′-CAG​TGC​GTG​TCG​TGG​AGT-3′.

### 2.3 Investigating the Novel Structure of iDNMT-1

#### 2.3.1 Molecular docking

All molecular docking experiments were performed using the crystal structure of DNMT1 obtained from the Protein Data Bank (PDB; PDB ID: 3PTA). DNMT1 was prepared in AutoDock Vina software by removing the ligand and water molecules, followed by the addition of intramolecular hydrogen bonds (Trott et al., 2010; Feng et al., 2020a; Feng et al., 2020b). The conformational search space was then defined as a cube centered on iDNMT-1 (x = 23.105; y = −19.538; Z = −29.942), with a side length of 24.158 Å. All docking simulations were run at the default settings, and the search accuracy was set to level 8. In total, nine docking models were generated under these conditions, and the maximum energy value differed from the optimal combination model is 3 kcal/mol. It took approximately 30 min for each docking simulation to run. All docked poses were visualized in PyMOL software [http://www.pymol.org].

#### 2.3.2 Lead Compound Validation

In this study, a compound library containing 170,000 molecules was downloaded from the ZINC database (http://zinc.docking.org). *In vitro* activity data are available for all molecules in this library. Subsequently, 72,000 compounds were screened in a preliminary screen using DruLiTo software (https://en.freedownloadmanager.org/Windows-PC/DruLiTo-FREE.html) using the following parameters: 0 < Moriguchi log P (MLogP) < 5, 250 < molecular mass <400, 1 ≤ hydrogen bond donors ≤5, 1 ≤ hydrogen bond acceptors ≤10, and 2 ≤ aromatic nucleus ≤4.

AutoDock Vina was used to screen the 72,000 compounds using the ligands in the original protein as the docking template. A total of 683 compounds were excluded because their docking scores were higher than those of the original ligands. Continuous fine screening was performed with AutoDock Vina to obtain the top 50 compounds which were then analyzed by cluster analysis. Next, the representative compounds with large structural differences were empirically selected. Finally, a total of 12 representative compounds were selected (see supporting document), purchased from Medrige or synthesized by ourselves. *In vitro* activity testing leads to the identification of N-(4-acetylphenyl)-3-methoxy-4-methylbenzamide (ZINC15602870) as the leading inhibitor of DNMT with a half-maximal inhibitory concentration (IC_50_) of 20 µM on DNMT-1.

#### 2.3.3 Lead Compound Optimization

To improve the activity of DNMT1 inhibitors, position R1 in ZINC15602870 was modified, and the groups with extended structure were transferred onto position R1 to generate compounds 02–04. *In vitro* activity data confirm that the aforementioned modifications in these three compounds (i.e., No. 02, No. 03, and No. 04) generated improved IC_50_ values compared to ZINC15602870, with compound No. 03 showing the highest affinity. Since the binding pocket in the crystal structure of DNMT1 is long and narrow, the two carbonyl structures on compound 03 allow for tighter protein–ligand interactions. A pyrrodiazole group was also introduced in the R1 position to generate compounds 05–09. Unlike compounds 02–04, compounds 05–09 contain a flexible side chain (Table 1). However*, in vitro* activity data showed that compounds 05, 06, 08, and 09 did not show improved activity, suggesting that, with the exception of compound 07, the flexible side chain structure does not provide any advantage for improved protein–ligand interactions. To improve hydrogen bonding between compounds and GLN1227, the structure of the 4-methoxy-3-methylbenzene group was modified by replacing the methyl in the original structure with a methoxy group (Table 1). The side chains of compounds 03 and 07 were then introduced onto position R2 of the new parent ring (Table 1). *In vitro* activity tests were then performed as previously described (Datta et al., 2009).

**TABLE 1:** Structure of compounds 01–11.

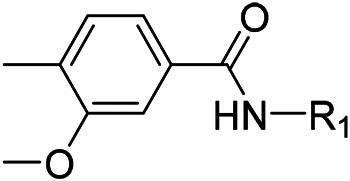		
Compound number	Structure	Activity (µM)
01	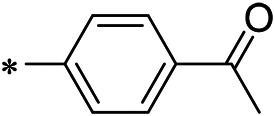	20.00
02	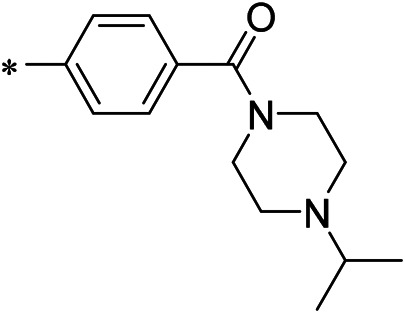	15.00
03	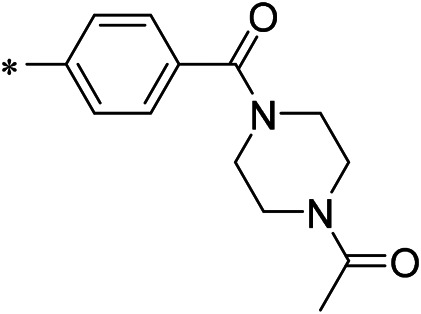	7.00
04	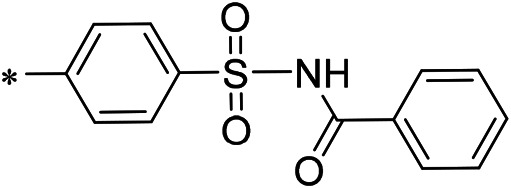	13.00
05	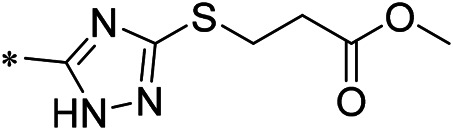	18.00
06	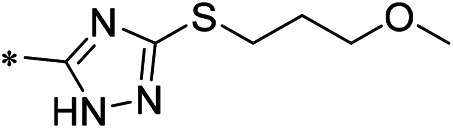	38.00
07	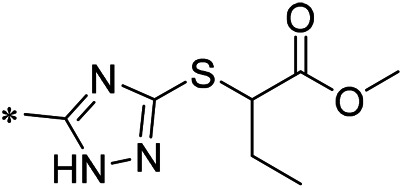	9.000
08	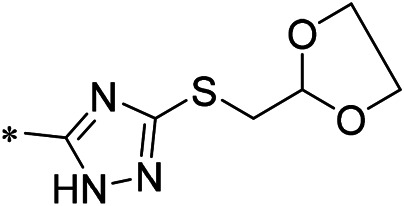	30.00
09	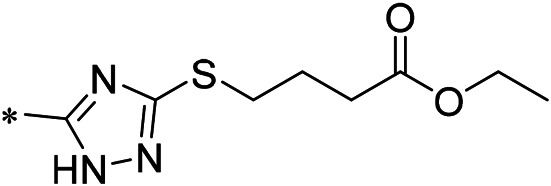	22.00
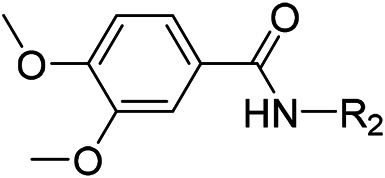		
10/iDNMT	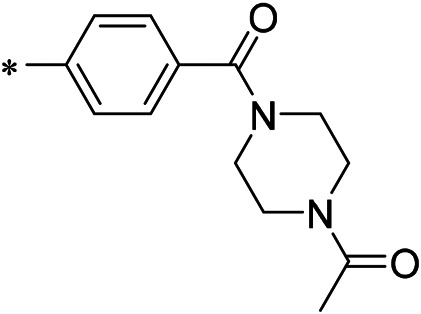	4.71
11	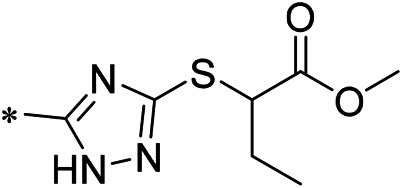	6.00
SGI-1027		5.38

### 2.4 Western Blotting and the Subcellular Fractionation

The H226 cells were cultured in Dulbecco’s modified Eagle’s medium (DMEM) with 10% (v/v) fetal bovine serum (FBS). The cells were transfected with plasmids or treated with the appropriate concentration of iDNMT [low dose (0.1 μmol/L), medium dose (1 μmol/L), and high dose (10 μmol/L)]. The H226 cells were then harvested for subcellular fractionation assays as previously described. The H226 cells were split into the nuclear subfraction or the cytoplasmic subfraction (Lu et al., 2013; Yang et al., 2013; Feng et al., 2018b). NICD, PSEN-1, and DNMT1 expression levels were examined in the subfractions by immunoblotting using antibodies from Santa Cruz (United States) or Abcam (United Kingdom). We chose β-actin and lamin A as the cytoplasmic and nuclear loading controls or indicators, respectively (Lu et al., 2013; Yang et al., 2013; Feng et al., 2018b).

### 2.5 RFA Treatment of LSCC Tissues

The antitumor efficacy of iDNMT, RFA, and iDNMT1 + RFA on LSCC tissues was examined in nude mice. Nude mice aged 5 weeks were obtained from the Si-Bei-Fu Corporation (Beijing, China), and they were fed under specific pathogen-free conditions. The H226 cells (approximately 5 × 10^6^ cells/animal), which were transfected with plasmids, were subcutaneously injected into nude mice to generate tumor tissues (Feng et al., 2019; Sun et al., 2019). The dimensions of these tumors were measured using a Vernier caliper every 7 days. Tumor volumes were calculated according to the following formula: tumoral width × tumoral width × tumoral length/2 (Feng et al., 2019; Sun et al., 2019). For RFA treatment or RFA + iDNMT treatment, RFA of the LSCC subcutaneous tumors was performed using a thyroid-ablation needle (Cat No.: UniBlate 700-103587 17G, RITA Company, Crystal Lake, IL, United States) when the tumoral volume reached ∼500 mm^3^. The conditions for RFA were as follows: 2 min RFA-duration with a series of different temperatures, 65, 60, or 55°C. In addition, iDNMT was administered orally at 10, 5, 2, or 1 mg/kg (Xie et al., 2018a; Xie et al., 2018b; Zhou et al., 2021). The formulation of iDNMT and the oral administration were performed as previously described (Zou et al., 2021a; Zou et al., 2021b; Wang et al., 2021c; Jie et al., 2021). Subcutaneous tumors were separated from the animals, and the weights of these tumors were measured using a precision-balance.

### 2.6 Statistical Analyses

The Statistical Package for the Social Sciences (SPSS) statistical software package (version 24.0, IBM Corp., Armonk, NY, United States) was used for all statistical analyses. T-tests were used to compare the expression levels between two categorical variables, and a *p*-value < 0.05 was considered to be statistically significant. The related expression level of genes is shown as a heat-map as described by Zhou *et al.* (2020) (Zhou et al., 2021).

## 3 Results

### 3.1 miR-27-3p Hypermethylation Induces Aberrant PSEN-1 Expression in LSCC Samples

**FIGURE 1:**
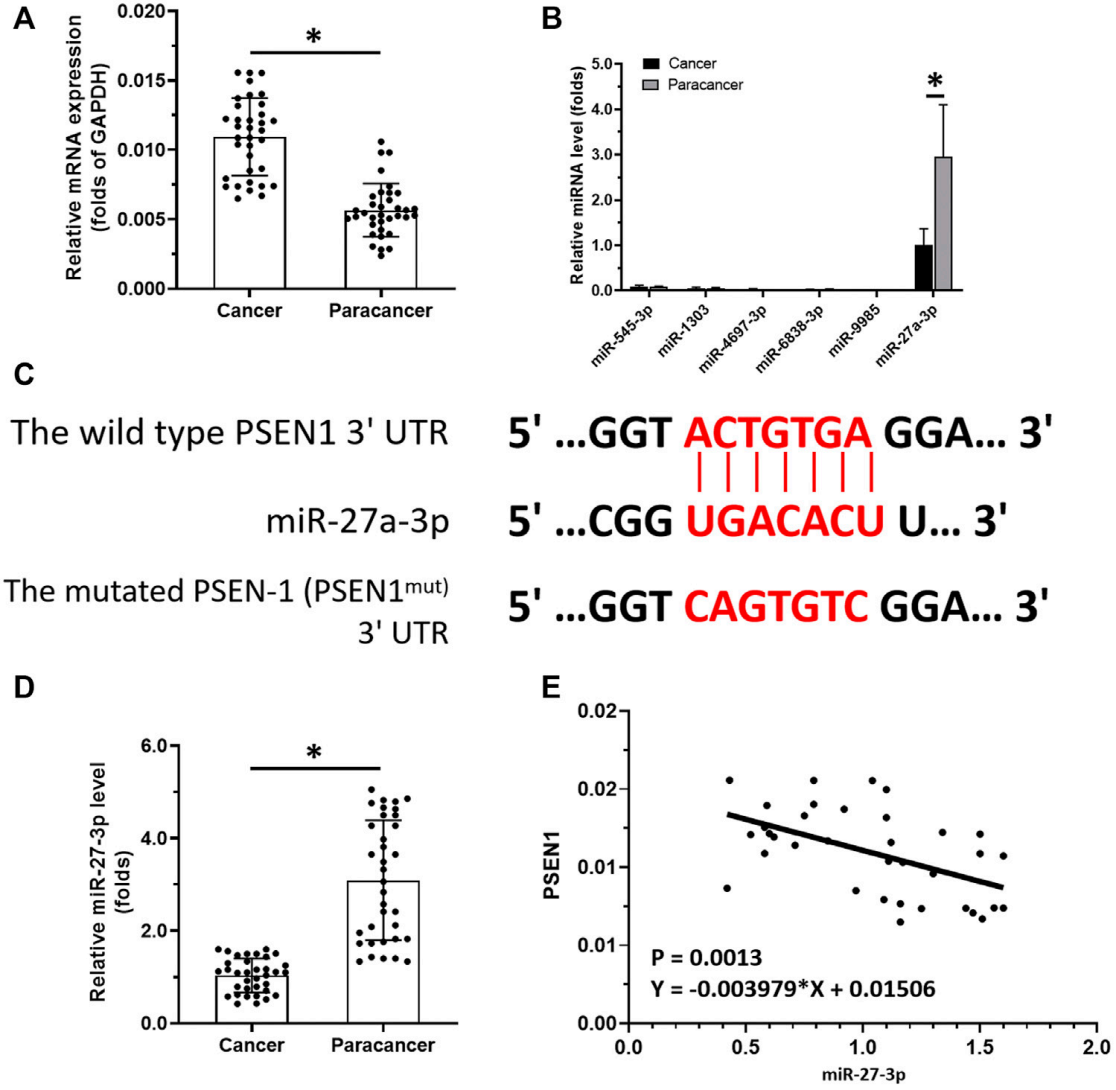
miR-27-3p/PSEN-1 axis in LSCC. **(A)** PSEN-1 expression in LSCC clinical specimens or the paired non-tumor tissues are illustrated as scatter-plots. **(B)** miRNA expression potentially targeting to PSEN-1 in LSCC or in the paired non-tumor tissues is shown as histograms. **(C)** Wild-type or the mutated sequences of miR27-3p-targeting sites located in the 3′UTR of PSEN-1. **(D)** miR-27-3p expression in LSCC clinical specimens is shown as a scatter-plot. **(E)** Correlation between PSEN-1 and miR-27-3p is depicted as a scatter-plot (*n* = 35). *p < 0.05.

To reveal the roles of Notch-related pathways in LSCC, we examined PSEN-1 expression in clinical specimens (i.e., the LSCC clinical specimens or the paired non-tumor tissues). We found that PSEN-1 expression was much higher in LSCC samples than paired non-tumor tissues (Figure 1A). To reveal the potential mechanisms behind this differential expression, we predicted potential PSEN-1-targeting miRNAs by qPCR. Among the selected miRNAs that may act on PSEN-1 (i.e., miR-545-3p, miR-1303, miR-4697-3p, miR-6838-3p, miR-9985, and miR-27-3p), miR-27-3p expression was much higher than other miRs in the clinical specimens (Figures 1B,C). The expression of miR-27-3p is much lower in LSCC specimens than that in the paired non-tumor tissues (Figure 1D). While PSEN-1 is the target of miR-27-3p (Figure 1C), its expression is negatively associated with miR-27-3p in LSCC specimens. Therefore, the downregulation of miR-27-3p may contribute to the high level of PSEN-1 expression in LSCC.

**FIGURE 2:**
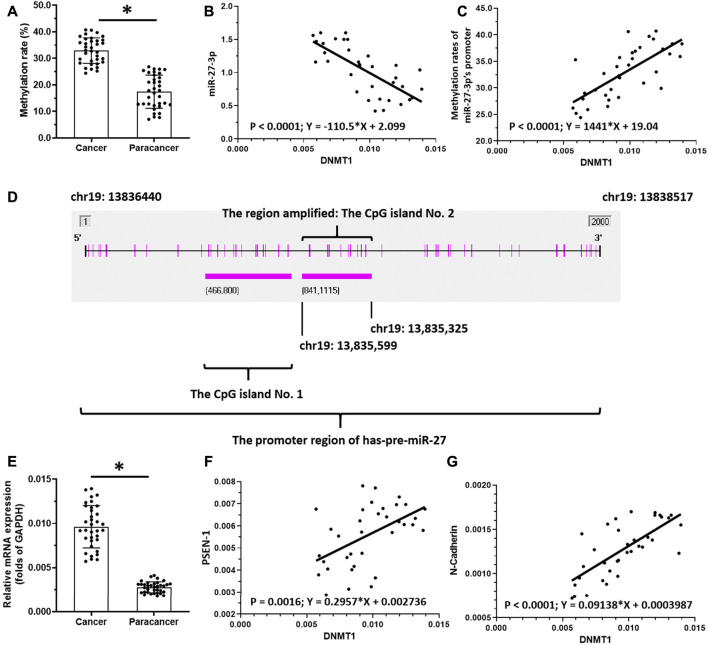
miR-27-3p hypermethylation. **(A)** Methylation rates of mR-27-3p in LSCC clinical specimens or the paired non-tumor tissues are shown as scatter-plots. **(B)** Correlation between miR-27-3p and DNMT-1 is shown as scatter-plot images. **(C)** Correlation between the methylation of miR-27-3p and DNMT-1 is shown as a scatter-plot. **(D)** Promoter region of miR-27-3p with CpG island. **(E)** DNMT-1 expression in LSCC or the paired non-tumor tissues is shown as a scatter-plot. **(F)** Correlation between PSEN-1 and DNMT-1 is shown as scatter-plots. **(G)** Correlation between N-cadherin and DNMT-1 is shown as scatter-plot (*n* = 35). *p < 0.05.

To reveal the potential mechanism of miR-27-3p downregulation in LSCC, we analyzed the methylation rates of the miR-27-3p promoter (Figures 2A–D). Interestingly, our data show that the methylation rates of the miR-27-3p promoter are much higher in LSCC specimens than those of the paired non-tumor tissues (Figure 2A). Next, we examined the association with DNMT-1, which mediates the methylation rates of the miR-27-3p′s promoter region, with the miR-27-3p/Notch pathway. These data showed that DNMT-1 expression is negatively associated with miR-27-3p expression while being positively associated with methylation of the miR-27-3p promoter region (Figures 2B,C ). Moreover, DNMT-1 expression was much higher in LSCC specimens than that in the paired non-tumor tissues (Figure 2E) and was positively correlated with PSEN-1 (Figure 2F) and N-cadherin expression, typical downstream factors of Notch pathway-related to EMT (Figure 2G). Furthermore, Figure 2D suggests that the promoter region of miR-27-3p contains CpG islands. Therefore, DNMT-1 may mediate the downregulation and upregulation of miR-27-3p and PSEN-1 expression, respectively, by inducing hypermethylation of the miR-27-3p promoter in LSCC.

### 3.2 iDNMT-1 Inhibits Notch Pathway Activation via DNMT-1/miR-27-3p

**FIGURE 3:**
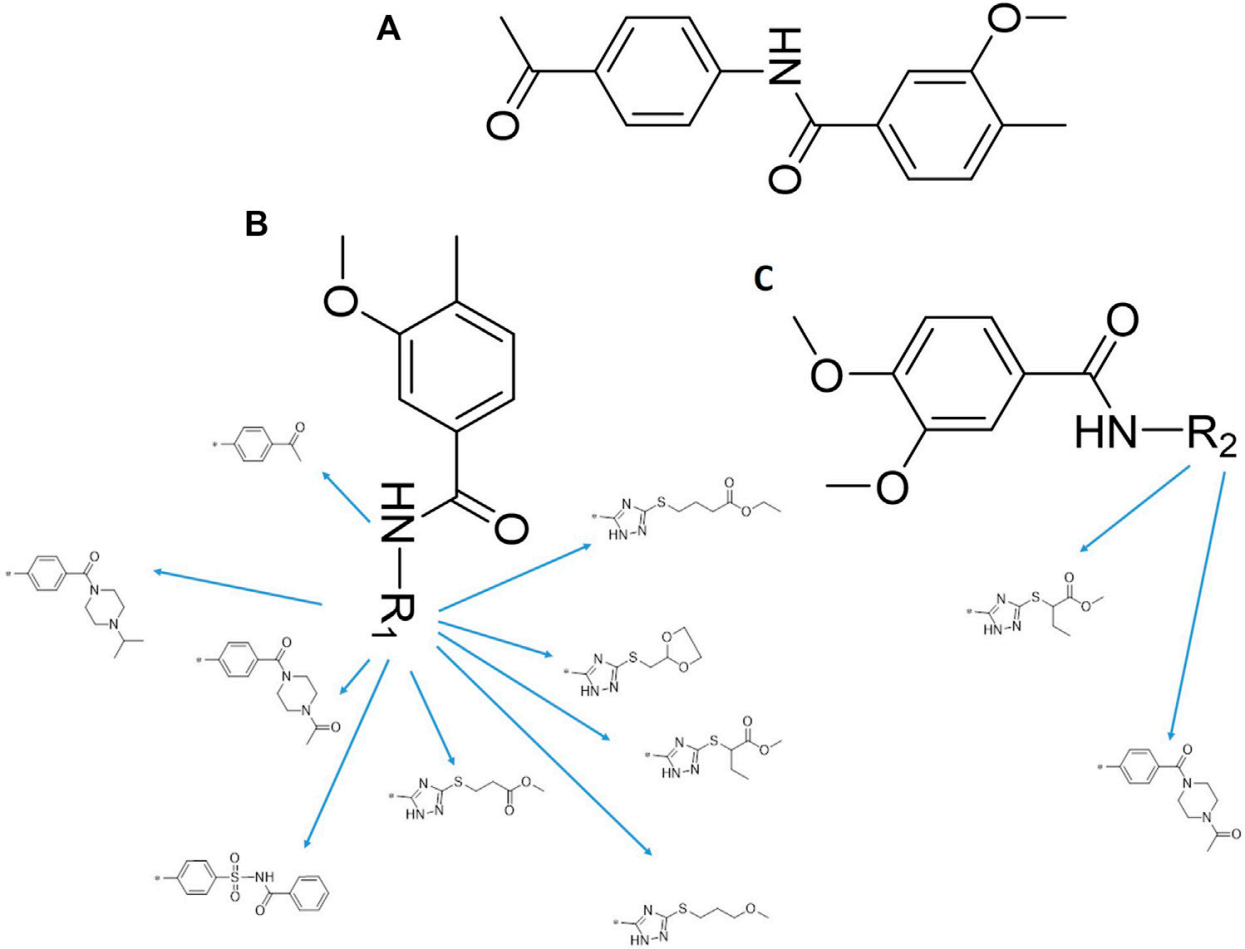
Leading inhibitory DNMT-1 compound. **(A)** Structure of the leading compound. **(B,C)** R1 and R2 positions for leading compound substitutions.

N-(4-acetylphenyl)-3-methoxy-4-methylbenzamide was chosen as the leading compound following virtual screening experiments (Figure 3A). This compound (mentioned in Figure 3A) was modified at positions R1 (Figure 3B) and R2 (Figure 3C) to obtain the lead compound. The generated compound series is shown in Table 1. Compound 10 (iDNMT) was selected as the lead compound because it showed the most superior activity.

**FIGURE 4:**
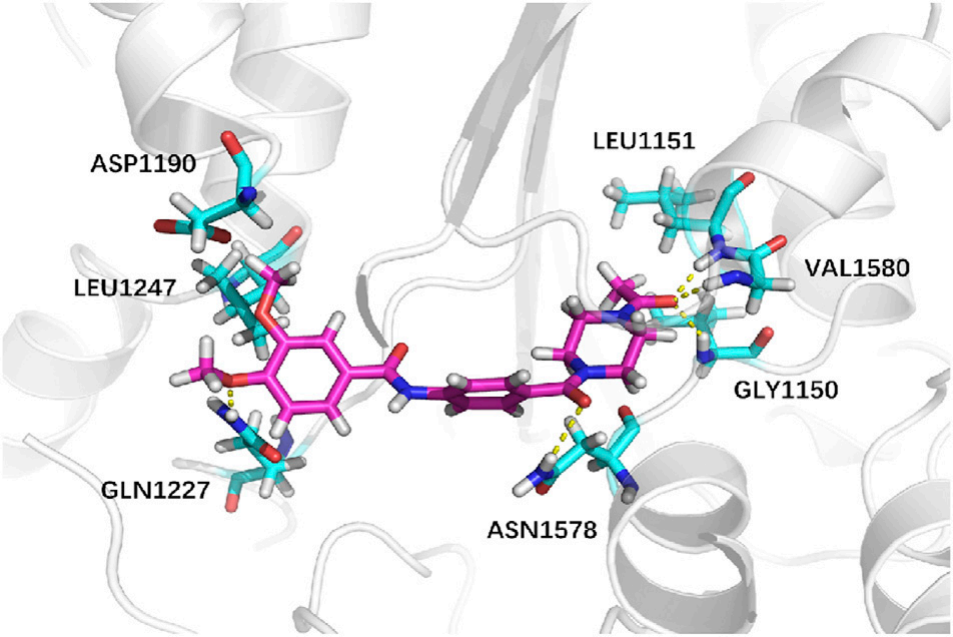
Predicted mode of iDNMT/DNMT-1 binding.

Next, we explored virtual docking as a tool to elucidate the mechanism of binding between iDNMT and DNMT-1. The molecular size of iDNMT1 is such that it fits almost exactly in the cavity of the combined pocket in DNMT-1. The dimethoxyphenyl structure of iDNMT1 stabilizes the configuration through hydrophobic interactions with DNMT-1 and forms a hydrogen bond with GLN1227 through the oxygen atom on the methoxy group. This explains why compound No. 10 is more active than compound 03. The carbonyl in the middle of the compound (No. 10) forms a hydrogen bond with ASN1578 with a bond length of 2.6 Å. The carbonyl at the end of the compound forms a hydrogen bond with GLY1150, LEU1151, and VAL1580 with bond lengths of 2.1, 1.9, and 2.3 Å, respectively. These strong hydrogen bonds explain why iDNMT is such an effective inhibitor of DNMT1 (Figure 4).

**FIGURE 5:**
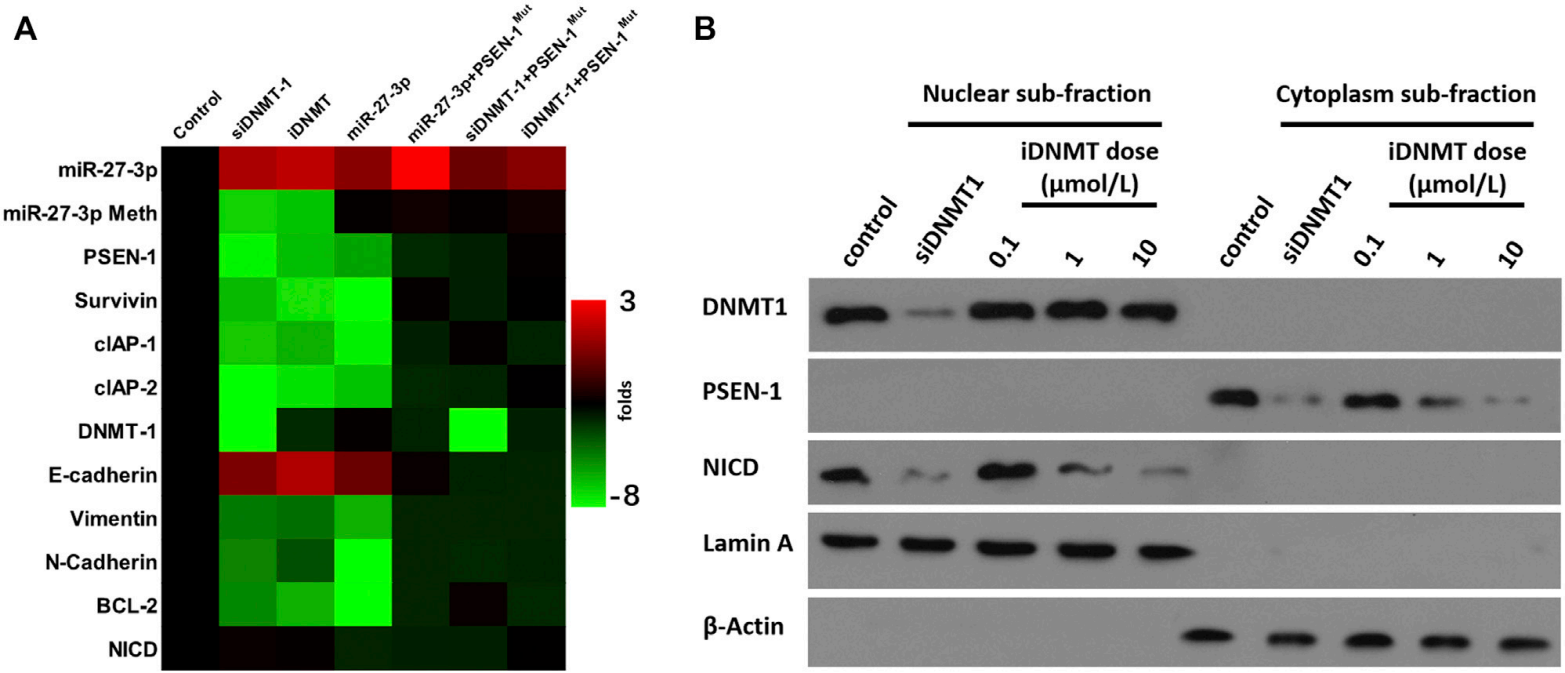
iDNMT inhibits activation of the Notch pathway. **(A)** Effect of iDNMT on the expression of Notch pathway-related factors is depicted as a heat-map. **(B)** Effect of iDNMT on Notch protein cleavage is shown as cellular subfraction.

The aforementioned results indicated that iDNMT could inhibit DNMT-1 activity. Therefore, the effect of iDNMT on the Notch pathway was further examined. The treatment of iDNMT increased miR-237-3p expression to inhibit the expression of PSEN-1 or downstream factors of the Notch pathway. These include the EMT-related factors (the N-cadherin or vimentin) or the pro-survival/antiapoptosis-related factors (urvivin, cIAP-1, or cIAP-2). Treatment with iDNMT1 also decreased methylation of the miR-27-3p promoter region but not DNMT-1 expression (Figure 5A). Therefore, these data clearly show that iDNMT inhibits activation of the Notch pathway via the DNMT-1/miR-27-3p/PSEN-1 axis.

### 3.3 Specificity of iDNMT on the Notch Pathway

To further confirm the specificity of iDNMT on the Notch pathway, we analyzed the cytoplasm–nuclear subfraction using immunoblotting. We found that DNMT-1 and NICD localized in the nuclear subfraction of the H226 cells, whereas the PSEN-1 localized in the cytoplasm subfraction of H226 cells (Figure 5B). Low, medium, and high dose treatment with iDNMT resulted in reduced accumulation of NICD in the nuclear subfraction of H226 cells in a dose-dependent manner. However, there were no effects on DNMT-1 expression. Treatment with iDNMT also inhibited PSEN-1 expression in H226 cells, but again this did not affect DNMT-1 expression (Figure 5B). Interestingly, transfection of the siRNA of DNMT-1 (siDNMT-1) control induced a similar effect as iDNMT on PSEN-1 and NICD but inhibited DNMT-1 expression in H226 cells (Figure 5B). The methylation rates of the miR-27-3p′s promoter region and expression level of DNMT-1 or miR-27-3p in cell samples of Figure 5B are shown in Supplementary Figure S1. Therefore, iDNMT represses PSEN-1 expression to inhibit cleavage of Notch, thus inhibiting NICD accumulation in H226 cells.

### 3.4 iDNMT Enhances the Antitumor Effects of RFA Treatment on LSCC in a Murine Tumor Model

**FIGURE 6:**
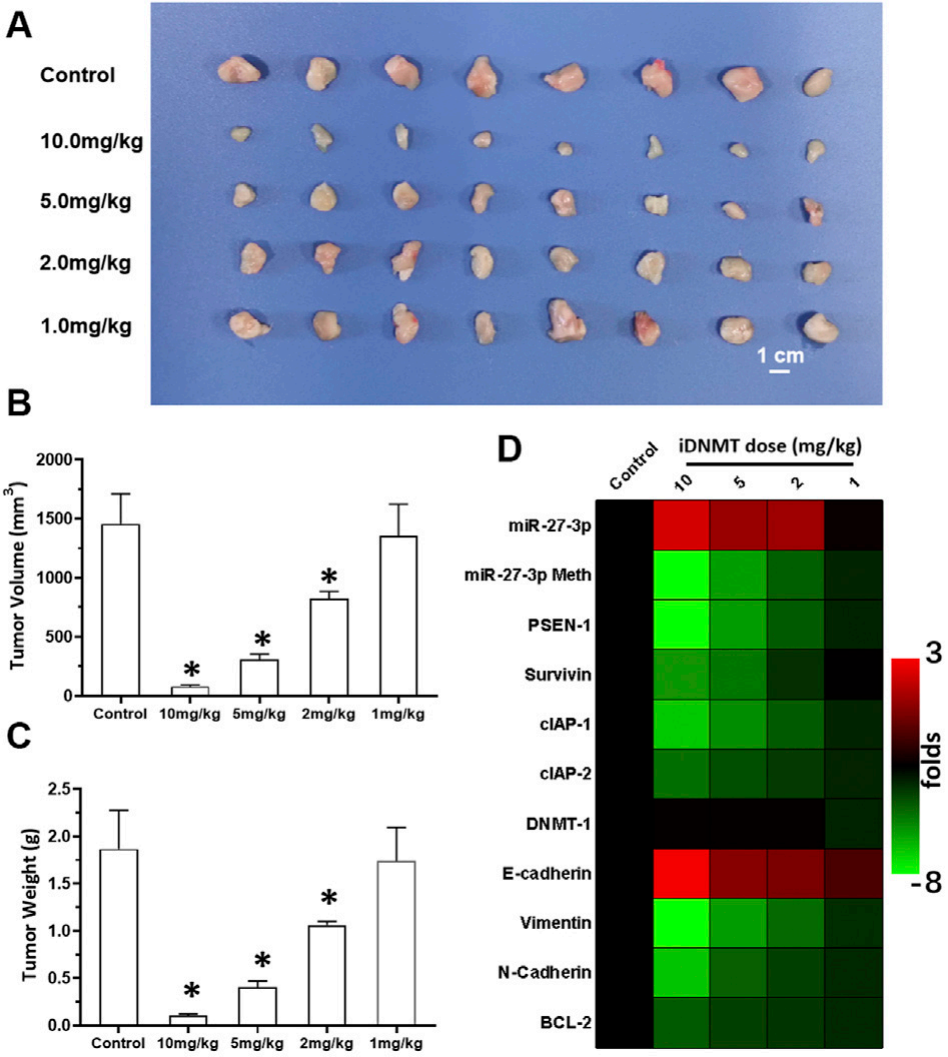
Oral administration of iDNMT inhibits the subcutaneous proliferation of H226 LSCC cells in nude mice. **(A)** Images of tumor tissues. **(B, C)** Volumes and weights of tumor tissues are displayed as histograms. **(D)** Effect of iDNMT on the expression of Notch pathway-related factors is displayed as a heat-map. Comparison between the iDNMT group and control group. *p < 0.05.

To examine the antitumor effect of combined iDNMT and RFA treatment on LSCC, we studied the effects of RFA and iDNMT under different conditions on LSCC. Oral administration of iDNMT alone inhibited the subcutaneous growth of H226 cells in nude mice with 10 mg/kg and 5 mg/kg doses showing the most significant inhibition (Figure 6). The 2 mg/kg dose of iDNMT alone had weak antitumor activity (cytotoxicity); however, at this dose, iDNMT significantly upregulated miR-27-3p expression leading to inhibition of the Notch pathway (i.e., downregulation of PSEN-1 expression and Notch downstream EMT, and cell pro-survival- and antiapoptotic-related factors; Figure 6). Therefore, we used 2 mg/kg dose of iDNMT for all subsequent experiments.

**FIGURE 7:**
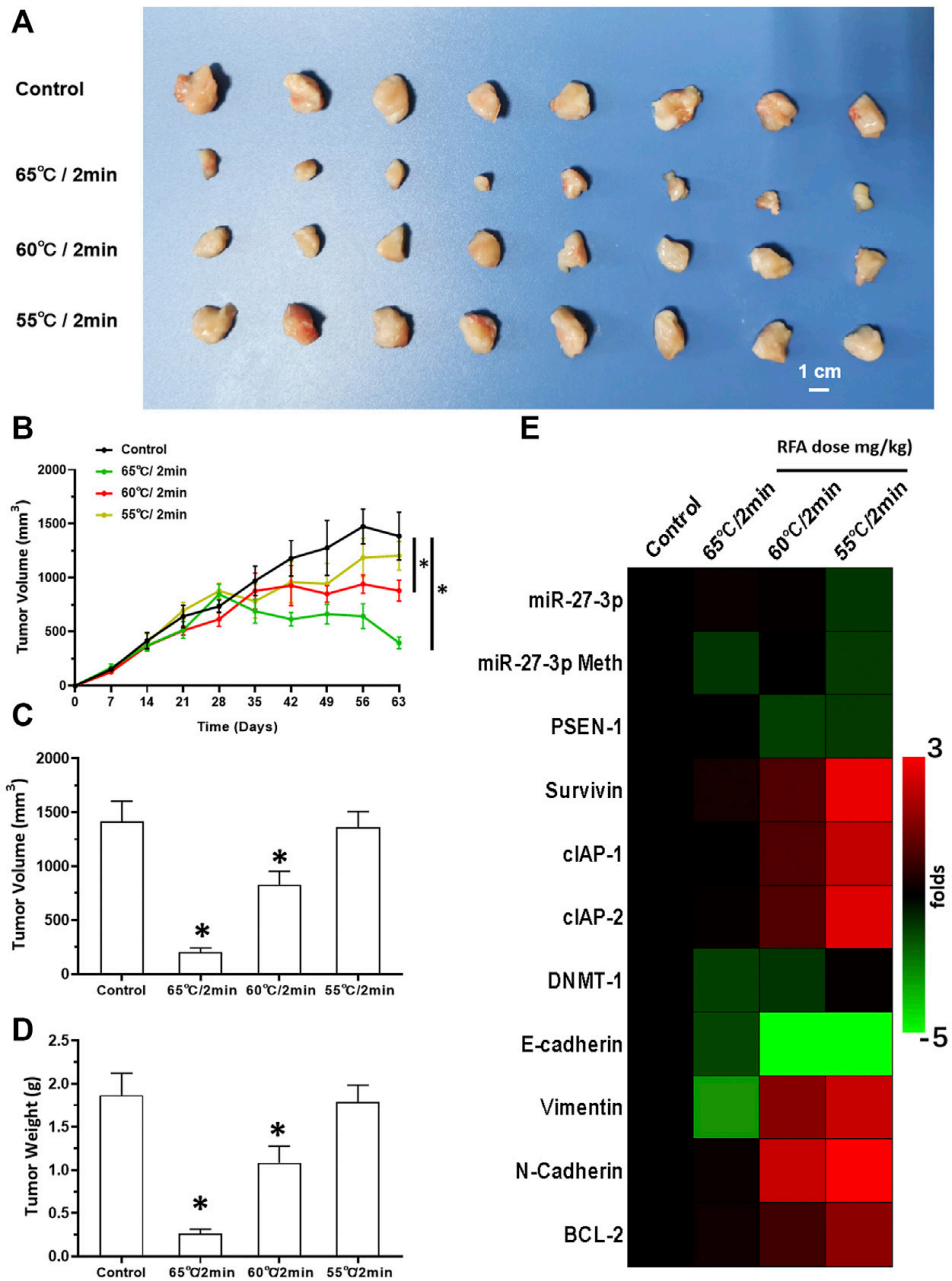
RFA treatment inhibits subcutaneous growth of H226 LSCC cells in nude mice. **(A)** Images of tumor tissues. **(B)** Tumor growth curves. **(C,D)** Volumes and weights of the tumor tissues are displayed as histograms. **(E)** Effect of RFA on the expression of Notch pathway-related factors is shown as a heat-map. Comparison between the iDNMT group and control group. *p < 0.05.

Next, we also studied the effect of RFA treatment on LSCC. The LSCC tissues were treated according to three different RFA conditions (65, 60, and 55°C all for 2 min). We found that treatment with RFA at 65°C significantly attenuated the proliferation of LSCC cells in nude mice (Figure 7). These data showed that volumes of subcutaneous tumors shrank gradually post RFA treatment (Figure 7). The antitumor effects observed at 60°C were much weaker than those of RFA treatment at 65°C. RFA treatment at 55°C did not show any significant inhibitory activation on LSCC cells, and these conditions induced the EMT process or the cellular injury response as characterized by the upregulation of the expression of related factors in LSCC tissues (Figure 7). Thus, 2 min RFA treatment at 55°C was shown to induce incomplete RFA therapy. This condition was chosen for the subsequent series of experiments.

**FIGURE 8:**
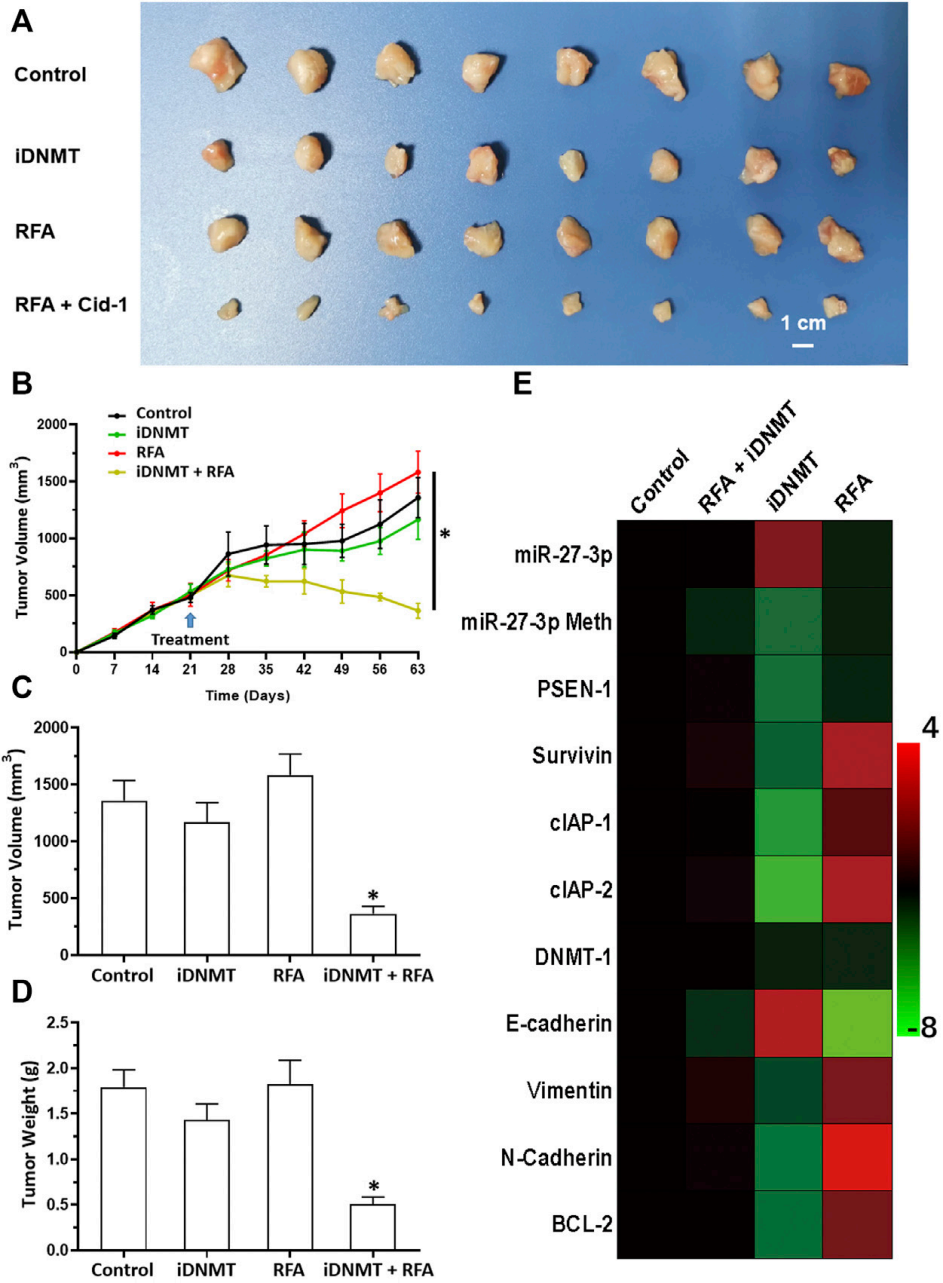
RFA + iDNMT inhibits the subcutaneous proliferation of H226 LSCC cells in nude mice. **(A)** Images of tumor tissues. **(B)** Tumor growth curves. **(C,D)** Volumes and weights of tumor tissues are displayed as histograms. **(E)** Effect of RFA or iDNMT on the expression of Notch pathway-related factors is displayed as a heat-map. Comparison between the iDNMT group and control group. *p < 0.05.

Next, we analyzed the effects of combined iDNMT/RFA-induced inhibition of LSCC cells. Our data showed that the combination treatment of LSCC cells with 2 mg/kg iDNMT with 2 min RFA treatment at 55°C significantly induced shrinkage of LSCC tumor volumes (Figure 8). Furthermore, iDNMT also inhibited the EMT process of LSCC cells in subcutaneous tissues, which we had previously observed following RFA treatment (55°C for 2 min; Figure 8). We show that iDNMT enhances the antitumor effect of RFA on LSCC, making combined iDNMT/RFA treatment a potentially promising strategy for LSCC treatment.

## 4 Discussion

**FIGURE 9:**
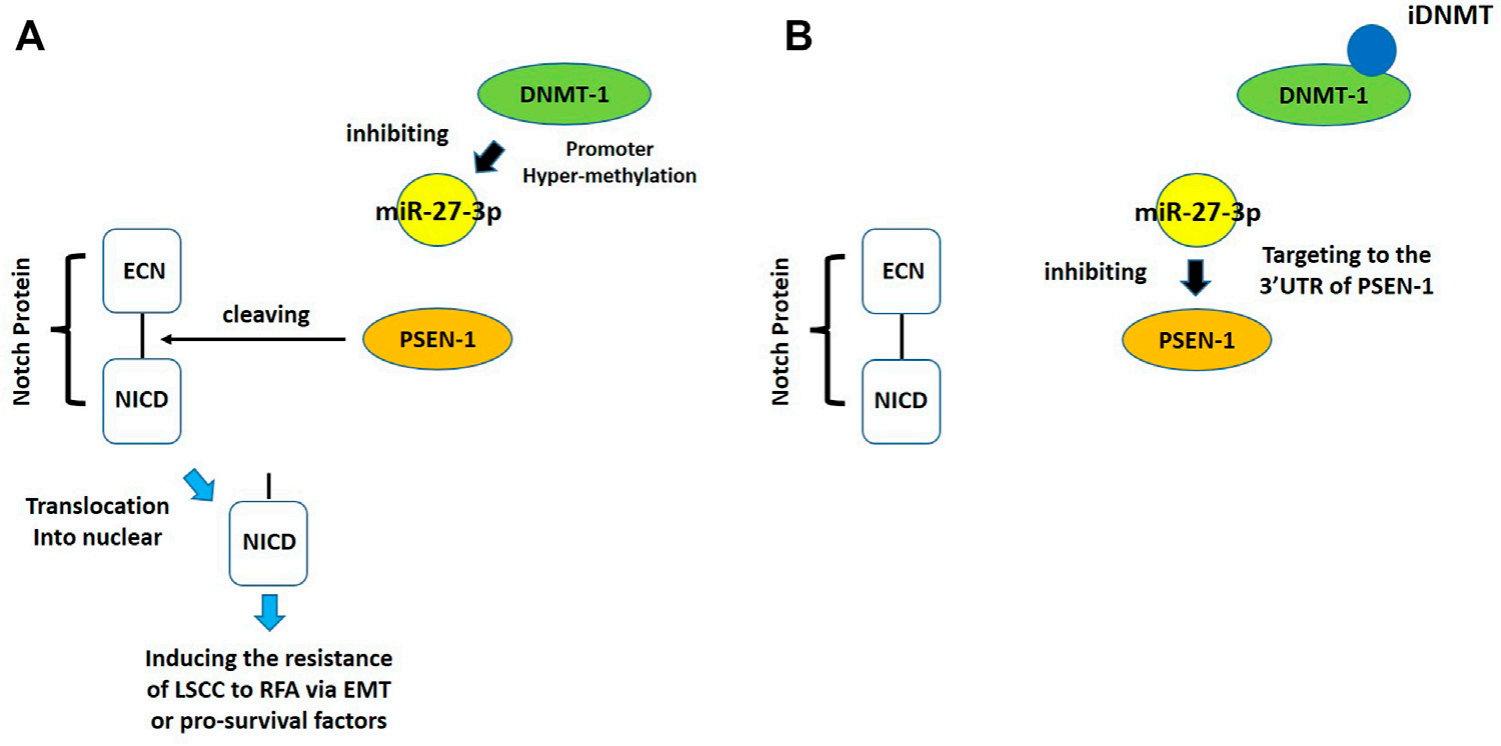
Proposed model of the present work. **(A)** In LSCC cells, DNMT-1 may induce cleavage and methylation of the miR-27-3p promoter, thereby inhibiting expression of miR-27-3p. Low miR-27-3p expression leads to upregulated PSEN-1 expression, which activates the Notch pathway by cleaving the Notch protein. **(B)** Treating LSCC cells with iDNMT inhibits DNMT-1 activity, downregulates methylation of the miR-27-3p promoter region, upregulates miR-27-3p expression, downregulates PSEN-1 expression, and ultimately inhibits Notch pathway activity.

Despite the recent advances in LSCC research, there are still no target specific pharmacological agents for LSCC treatment (Yao et al., 2021). This is a significant unmet need that must be explored to develop novel therapeutic strategies to improve the diagnosis, prevention, and treatment of LSCC. As well as being an epigenetic mechanism, hypermethylation of CpG islands in the promoter region of tumor suppressors (e.g., miR-27-3p) also contributes to the development of antitumor drug resistance (Goossens et al., 2019; Jiao et al., 2019; Qi et al., 2019). In mammalian cells, DNA-methylation is mediated by four types of DNA methyltransferases (i.e., DNMT-1, DNMT-2, DNMT-3a, and DNMT-3b) (Gutiérrez et al., 2021; Wang et al., 2021d; Wu et al., 2021). Aberrant expression/overexpression of DNMT-1 is an important regulator in the progression of human malignancies, acting to induce the silencing of tumor suppressors by mediating methylation of their promoters (Shayeghan et al., 2021; Yadav et al., 2021). In this work, we identified iDNMT, a small molecule inhibitor of DNMT-1 with a unique pharmacophore (Figure 3; Table 1 and Figure 9). Treatment with iDNMT inhibits DNMT-1 activation to repress methylation of the miR-27-3p promoter region (Figure 9). This leads to reduced activation of the Notch pathway by enhancing miR-27-3p expression (Figure 9). Currently, there are still many unresolved issues with application of DNMT-1 inhibitors, namely, there are currently DNMT-1 inhibitors in clinical use and the preclinical testing of the DNMT-1 inhibitor, SGI-1027, which has revealed that its activity is far from satisfactory. Specifically, the affinity of SGI-1027 for DNMT-1 is insufficient (She et al., 2020; Hong et al., 2021; Gao et al., 2022), and it has an IC_50_ value of ∼6 μmol/L (Feng et al., 2020b). The activity of iDNMT in this study is significantly better than that of SGI-1027, thereby providing additional insights into DNMT-1 inhibitors and expanding potential therapeutic options for LSCC treatment

As an interventional treatment strategy, RFA has been widely used in the treatment of malignant tumors (Fan et al., 2021; Bhandal et al., 2022). RFA is currently mostly used in the treatment of internal organs such as HCC (hepatocellular carcinoma) rather than for organs with cavities (e.g., lungs) which presents the following challenges: 1) at present, the RFA’s implementation strategy mainly relies on transdermal puncture using medical imaging (i.e., CT scan). As such, if RFA is to be applied to treat lung cancer, the direction of the puncture will be affected by the ribs; 2) RFA-induced tumor tissue damage occurs mainly by thermal incarnation, which risks pulmonary blood flow and potential lung ventilation disorders because RFA is too intense for lung tissue; and 3) lung tissues have an abundant supply of capillaries, and because RFA may damage the microcirculation this can endanger a patient’s normal breathing. This necessitates the development of safer and more effective RFA treatment strategies and/or combined RFA/drug treatment strategies. This would allow for the use of more moderate RFA conditions enabling equivalent or even more effective antitumor outcomes. In this study, we found that relatively mild, a 2 min RFA treatment at 55°C also simulates incomplete RFA. Importantly, we also confirmed that iDNMT enhances the killing effect of RFA on LSCC tissues, which in combination with the aforementioned milder RFA conditions, can achieve effective antitumor effects. EMT is closely related to tumor recurrence after RFA treatment (Dong et al., 2015; Iwahashi et al., 2016; Zhang et al., 2019; Zhou et al., 2019; Zhou et al., 2020). Encouragingly, iDNMT also inhibits the EMT of tumor tissues caused by incomplete RFA ablation. These data demonstrate that combination of iDNMT/RFA treatment may be a safer and more effective treatment strategy for LSCC.

The Notch pathway is an important regulator of tumor cell drug resistance (Schenk et al., 2021; Zhang et al., 2022). There is increasing evidence to suggest that inhibition of the Notch pathway can upregulate the sensitivity of malignant tumor cells to radiotherapy and antitumor drugs (Yang et al., 2021). The results of this study indicate that inhibition of PSEN-1 expression by miR-27-3p downregulates the activity of the Notch pathway by inhibiting the cleavage of the Notch protein, thereby increasing sensitization of LSCC to RFA (Zhao et al., 2021). These data provide important insights into what we already know about the Notch signaling pathway. In addition to chemotherapy and radiotherapy, RFA is now an important strategy in anticancer therapy. For RFA of LSCC, Zhou et al. (2020) found that combined treatment with RFA and anlotinib, the latter is implicated in inhibition of the EMT of LSCC, leading to an improved RFA antitumor effect (Zhou et al., 2021). However, while molecular-targeted drugs such as anlotinib and sorafenib induce sensitization to RFA or radiotherapy by inhibiting the EMT of malignant tumor cells, these molecules do not inhibit EMT as iDNMT does (Roskoski, 2020; Roskoski, 2021; Roskoski, 2022). The most specific molecular mechanism of the targeted drugs in this study, and one of our most important findings, is the induction of EMT in malignant cells (Chen et al., 2020). Therefore, we conclude that iDNMT is an important pharmacological tool for downregulating PSEN-1 expression via miR-27-3p, thereby inhibiting the cleavage and activation of the Notch protein, thus inhibiting the EMT of LSCC cells.

There are many different strategies for inhibiting the Notch pathway. For example, Zhao and his colleagues used small molecule inhibitors to inhibit ADAM17 activity to inhibit Notch protein cleavage (An et al., 2017; Li et al., 2018; Zhang et al., 2018; Lu et al., 2019; Lu et al., 2020). First-step cleavage of the Notch protein is mediated by ADAMs, while second-step cleavage is mediated by the complex multi-subunit protein, γ-secretase (Jia et al., 2021b). The latter is more important in the context of this work because the catalytic subunit PSEN-1 is the basis for γ-secretase activity (Jia et al., 2021b). Jia et al. (2021) systematically summarized known small molecule inhibitors that act on PSEN-1 and γ-secretase (Jia et al., 2021b). Zhao et al. (2021) found that miR-27-3p inhibits the activity of the Notch pathway in TNBC cells to upregulate TNBC cell sensitivity to olparib (Zhao et al., 2021). A common feature of these data is inhibition of ADAM or γ-secretase leads to inhibition of Notch protein cleavage. It is worth mentioning that Zhao et al. (2021) prepared miR-27-3p (that is, using the full sequence of hsa-pre-miR-27) as a lentiviral vector to inhibit PSEN-1 expression (Zhao et al., 2021). Here, we first found that hypermethylation of miR-27-3p in the promoter region of LSCC tissue leads to its downregulation. We further used iDNMT to induce pharmacological downregulation of miR-27-3p expression. Methylation of the promoter region upregulates miR-27-3p expression leading to increased sensitivity of LSCC to RFA. These data provide novel insights that extend our knowledge of PSEN-1 biology. It is worth mentioning that in addition to Notch protein cleavage, Kang *et al.* (2013), Jia *et al.* (2016), Li *et al.* (2021), and Ma *et al.* (2020) used various strategies to upregulate miR-34a in different malignant tumor tissues such as HCC and NSCLC to inhibit Notch protein expression (Kang et al., 2013; Jia et al., 2016; Ma et al., 2020; Li et al., 2021) to upregulate the sensitivity of malignant cells to antitumor strategies. Ma *et al.* (2020) also found that the promoter region of miR-34a was hypermethylated in pancreatic cancer mediated by DNMT-1. Also, downregulation of DNMT-1 expression with siRNA could inhibit the methylation of the miR-34a promoter region. In the future, it will be important to determine miR-34a expression in LSCC and the extent of methylation at the miR-34a promoter region.

## Data Availability

The original contributions presented in the study are included in the article/Supplementary Material, further inquiries can be directed to the corresponding authors.
