# Supplementary figures and images for: A Novel Small Molecular Inhibitor of DNMT1 Enhances the Antitumor Effect of Radiofrequency Ablation in Lung Squamous Cell Carcinoma Cells
Source: Front Pharmacol. 2022 Mar 23;13:863339. doi: 10.3389/fphar.2022.863339 (PMC8983860; doi:10.3389/fphar.2022.863339)

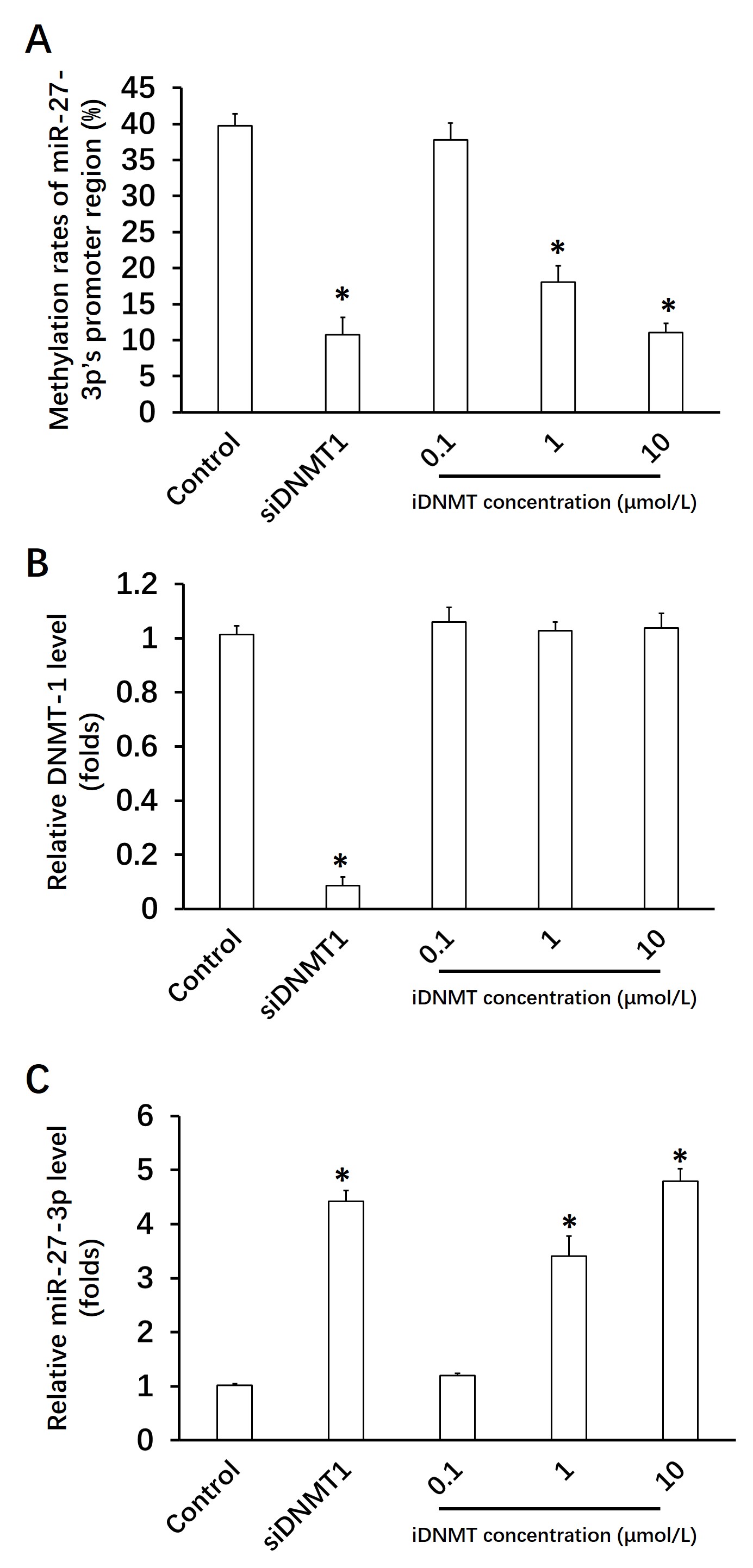

Supplement: Supplementary file 1 [file Image1.JPEG]
